# Variability in subpopulation formation propagates into biocatalytic variability of engineered *Pseudomonas putida* strains

**DOI:** 10.3389/fmicb.2015.01042

**Published:** 2015-10-01

**Authors:** Martin Lindmeyer, Michael Jahn, Carsten Vorpahl, Susann Müller, Andreas Schmid, Bruno Bühler

**Affiliations:** ^1^Laboratory of Chemical Biotechnology, Department of Biochemical and Chemical Engineering, TU Dortmund UniversityDortmund, Germany; ^2^Helmholtz Centre for Environmental Research - UFZ, Department for Environmental MicrobiologyLeipzig, Germany; ^3^Helmholtz Centre for Environmental Research - UFZ, Department of Solar MaterialsLeipzig, Germany

**Keywords:** phenotypic heterogeneity, clonal variability, intra-population variability, fluorescent reporter, flow cytometry, plasmid copy number, *Pseudomonas putida*, *alk*-regulatory system

## Abstract

Pivotal challenges in industrial biotechnology are the identification and overcoming of cell-to-cell heterogeneity in microbial processes. While the development of subpopulations of isogenic cells in bioprocesses is well described (intra-population variability), a possible variability between genetically identical cultures growing under macroscopically identical conditions (clonal variability) is not. A high such clonal variability has been found for the recombinant expression of the styrene monooxygenase genes *styAB* from *Pseudomonas taiwanensis* VLB120 in solvent-tolerant *Pseudomonas putida* DOT-T1E using the *alk*-regulatory system from *P. putida* GPo1. In this study, the oxygenase subunit StyA fused to eGFP was used as readout tool to characterize the population structure in *P. putida* DOT-T1E regarding recombinant protein content. Flow cytometric analyses revealed that in individual cultures, at least two subpopulations with highly differing recombinant StyA-eGFP protein contents appeared (intra-population variability). Interestingly, subpopulation sizes varied from culture-to-culture correlating with the specific styrene epoxidation activity of cells derived from respective cultures (clonal variability). In addition, flow cytometric cell sorting coupled to plasmid copy number (PCN) determination revealed that detected clonal variations cannot be correlated to the PCN, but depend on the combination of the regulatory system and the host strain employed. This is, to the best of our knowledge, the first work reporting that intra-population variability (with differing protein contents in the presented case study) causes clonal variability of genetically identical cultures. Respective impacts on bioprocess reliability and performance and strategies to overcome respective reliability issues are discussed.

## Introduction

The use of whole-cell biocatalysts is a promising approach for applications in industrial biotechnology and is favored over the use of isolated enzymes, when host intrinsic cofactor regeneration, degradation of reactive oxygen species, and/or continuous synthesis of instable enzymes are required (Schmid et al., 2001; Woodley, 2006; Leak et al., 2009; Faber, 2011; Schrewe et al., 2013). The still limited understanding of intracellular regulatory network operation and causes for variability in isogenic microbial cultures often restricts industrial applications (Kuhn et al., 2010a). The overall productivity and stability of a bioprocess strongly relies on the physiology and metabolic performance of the microbial host. A major critical aspect is the occurrence of phenotypically diverse cells or subpopulations (described by the term “phenotypic heterogeneity”), which contribute differently to the production, conversion, or degradation of a desired product or substrate and thus hampering process efficiency (Achilles et al., 2007; Delvigne and Goffin, 2014). Thereby, variations in protein contents or variable expression patterns among different cells in an isogenic population appear (Elowitz et al., 2002; Fraser and Kaern, 2009; Jahn et al., 2012). Phenotypic heterogeneity often arises from stochastic variations in regulatory network operation and can be linked to variations in the amounts of intracellular compounds involved in gene expression such as transcription factors or inhibitors (Ryall et al., 2012). These variations are attributed to two different types of noise: extrinsic and intrinsic noise (Elowitz et al., 2002). Extrinsic noise arises from differing predispositions regarding the availability of molecules with global relevance for regulatory network operation, such as biomolecules involved in the biomass synthesis machinery (ribosomes, DNA-polymerases, RNA-polymerases). In contrast, intrinsic noise does not depend on regulatory predispositions but results from stochastic events, e.g., the frequency of collision/contact between the individual functional units involved in gene expression (inducer, promotor, regulator). Both intrinsic and extrinsic noise jointly contribute to phenotypic heterogeneity, whereas extrinsic noise was shown to make a greater contribution to total gene expression variability than intrinsic noise (Elowitz et al., 2002; Kaern et al., 2005; Avery, 2006).

Bimodality resulting from differential gene expression is a well described phenomenon (Novick and Weiner, 1957; Veening et al., 2006). Simplified, after induction of a certain regulatory system, one subpopulation contains a low level of a distinct protein signaling an OFF-state, whereas another subpopulation surpasses a threshold in the respective protein level signaling an ON-state. Resulting subpopulations and respective dynamics can be detected and characterized via flow cytometry in combination with protein marker molecules (Diaz et al., 2010; Müller et al., 2010). In contrast to bimodality, where cells can fluctuate between two states depending on the threshold signal concentration for activation, bistability describes a binary system in which the state of the regulatory system is maintained even after the trigger input is gone (Nikel et al., 2013). Such bistability can also result from genetic heterogeneities, e.g., caused by instable plasmid proliferation (Bentley and Quiroga, 1993). We recently have developed a method for the accurate quantification of the plasmid copy number (PCN) in subpopulations differently expressing a fluorescence marker gene (Jahn et al., 2014). For *Pseudomonas putida* KT2440, the bistability in protein production could be directly attributed to plasmid loss of a large fraction of cells resulting in genetic variability.

Regarding phenotypic heterogeneity, two types of variability can be considered: “intra-population variability” characterized by the development of phenotypically diverse subpopulations within a single isogenic culture and “clonal variability” describing the variability between individual isogenic cultures operated under macroscopically identical conditions.

Common examples for intra-population variability in bioprocessing include the appearance of cells with altered physiological properties in large scale fed-batch bioreactors (Enfors et al., 2001) or the development of a “non-producing” subpopulation under production conditions (Alonso et al., 2012). Most studies addressing phenotypic heterogeneity focus on intra-population variability, its characterization, and respective causes. Only few microbiological studies have targeted clonal variability and its relation to intra-population variability. In case of mammalian cells such as Chinese hamster ovary (CHO) cells used for recombinant gene expression, clones tend to differ markedly in expression after transfection due to variable gene copies in the chromosome or random integration. The occurrence of high producer clones after transfection is rare and respective screening and isolation represents a considerable challenge for the development of industrial applications (Pilbrough et al., 2009). However, the emergence of clonal variability is not restricted to mammalian cells, but also occurs in bacteria for plasmid-based heterologous gene expression. In a previous study, a high clonal variability in recombinant oxygenase levels has been detected for *P. putida* DOT-T1E and S12 but not for *P. putida* KT2440, *Pseudomonas taiwanensis* VLB120, and *E. coli* JM101 (Lindmeyer et al., 2015), when expression was based on the *alk*-regulatory system from *P. putida* GPo1 (van Beilen et al., 1994; Staijen et al., 1999). This variability was found not to depend on the type of heterologous enzyme and substrate/product nor on inducer toxicity or antibiotic resistance mechanisms. However, such clonal variability did not occur, when the *lac*-regulatory system was used. Thus, the interaction between the host-specific regulatory network and the regulatory system used for recombinant gene expression appeared to play a crucial role, giving evidence for an *alk*-regulatory system-related extrinsic type of noise as the cause for the observed clonal variability (Lindmeyer et al., 2015).

In this study, we investigated if such clonal variability in recombinant gene expression involves a variable subpopulation structure or relies on a random distribution of a large range of expression levels among individual cells, and if and to what extent such variability is related to PCNs. For this purpose, the styrene monooxygenase (StyAB) of *P. taiwanensis* VLB120 was chosen as model system. StyAB is composed of an oxygenase (StyA) and a reductase (StyB) component. Whereas, StyB catalyzes the transfer of electrons from NADH to freely diffusible FAD, StyA makes use of resulting FADH_2_ to reductively activate molecular oxygen and catalyze the epoxidation of styrene and derivatives to corresponding (*S*)-epoxides at a high enantiomeric access (*ee*) of ≥99% (Otto et al., 2004). Plasmid-based expression of a *styA-eGFP* gene fusion (Jahn et al., 2014) was studied regarding specific styrene epoxidation activity and specific fluorescence involving flow cytometry. The host strains *P. taiwanensis* VLB120, *E. coli* JM101, *P. putida* DOT-T1E, and *P. putida* KT2440 and the *alk*-, *tac*-, and *lac*-regulatory systems were used for *styA-eGFP* tool validation. For analyzing the interrelation of clonal and intra-population variability, the *alk*- and *lac*-regulatory systems were compared in *P. putida* DOT-T1E.

## Materials and methods

### Bacterial strains, plasmids, media, and chemicals

Unless otherwise stated, all chemicals and solutions were purchased from Sigma Aldrich (Steinheim, Germany) in the highest purity available. Bacterial strains and plasmids used in this study are listed in Table 1.

**Table 1:** **Bacterial strains and plasmids used**.

**Strain**	**Characteristics**	**References**
*E. coli* DH5α	*supE*44 Δ*lacU*169 (Φ80 *lacZ* ΔM15) *hsdR*17 *recA*1 *endA*1 *gyrA*96 *thi*-1 *relA*1	Hanahan, 1983
*E. coli* JM101	*supE thi* Δ(*lac-proAB*) F'[*traD36 proAB^+^ lac*I^q^ *lacZ* ΔM15]	Messing, 1979
*P. taiwanensis* VLB120	Wild-type, pSTY, Tol^r^	Köhler et al., 2013
*P. putida* DOT-T1E	Wild-type, pGRT1, Tol^r^, Rif^r^	Ramos et al., 1995
*P. putida* KT2440	mt-2 pWW0 cured, Tol^s^	Bagdasarian et al., 1981
**Plasmid**	**Characteristics**	**References**
pStyAB	pCom10, *alk*-regulatory system (*alkS*/P*_*alkB*_*), *styAB,* Km^r^,	Lindmeyer et al., 2015
pStyAB_lac	pCom10-derived, *lac*-regulatory system (*lac*I, P*_*lac*_*_*UV*5_), *styAB,* Km^r^	Lindmeyer et al., 2015
pStyAB_tac	pCom10-derived, *tac*-regulatory system (*lac*Iq, P*_*tac*_*), *styAB,* Km^r^	This study
pA-EGFP_B	pCom10, *alk*-regulatory system (*alkS*/P*_*alkB*_*), *styA-eGFP* and *styB,* Km^r^	Jahn et al., 2014
pA-EGFP_B _lac	pCom10-derived, *lac*-regulatory system (*lac*I, P*_*lac*_*_*UV*5_), *styA-eGFP* and *styB,* Km^r^	This study
pA-EGFP_B _tac	pCom10-derived, *tac*-regulatory system (*lac*Iq, P*_*tac*_*), *styA-eGFP* and *styB,* Km^r^	This study
pLac_alaD_ω-TA	Source of (*lac*I, P*_*lac*_*_UV5_)	Unpublished
pTAC:LS:AGPPS2	Source of (*lac*Iq, P*_*tac*_*)	Willrodt et al., 2014

*Km^r^, kanamycin resistance; Tol^r^, toluene resistance; Tol^s^, toluene sensitive; Rif^r^, rifampicin resistance*.

Bacteria were either grown on lysogeny broth (Bertani, 1951) composed of 5 g L^−1^ yeast extract (Difco, Detroit, USA), 5 g L^−1^ NaCl, and 10 g L^−1^ Tryptone (Oxoid, Hampshire, United Kingdom) or on minimal M9^*^ medium (25.5g L^−1^ Na_2_HPO_4_∙2H_2_O, 9.0 g L^−1^ KH_2_PO_4_, 1.0 g L^−1^ NH_4_Cl, 0.5 g L^−1^ NaCl) supplemented with 0.2% (v/v) 1M MgSO_4_ and 0.1% (v/v) US^*^ trace elements solution (4.87 g L^−1^ FeSO_4_∙7H_2_O, 4.12 g L^−1^ CaCl_2_∙7H_2_O, 1.5 g L^−1^ MnCl_2_∙4H_2_O, 1.87g L^−1^ ZnSO_4_, 0.84 g L^−1^ Na_2_EDTA∙2H_2_O, 0.3 g L^−1^ H_3_BO_3_, 0.25 g L^−1^ Na_2_MoO_4_∙2H_2_O, 0.15 g L^−1^ CuCl_2_∙2H_2_O and 82.81 mL 37% fuming HCl). For cultivations with *E. coli* M9^*^ medium was complemented with 0.5% (w/v) glucose. For the cultivation of *Pseudomonas* strains in M9^*^ medium, 0.5% (w/v) citrate was used as carbon source to avoid glucose-mediated catabolite repression of the *alk*-regulatory system (Staijen et al., 1999). M9^*^ medium was adjusted to pH 7.4 using NaOH and, if necessary, supplemented with 50 μg mL^−1^ kanamycin and 10 mg L^−1^ thiamine.

### DNA manipulation

Agarose gel-electrophoresis and ligation was performed as described by Sambrook and Russel (2001). Enzymes (Phusion High-Fidelity polymerase, T4 DNA ligase, restriction enzymes) and buffers were purchased from Thermo Scientific (St. Leon-Rot, Germany) and oligonucleotides from Sigma Aldrich (Steinheim, Germany). Plasmids and DNA-fragments were isolated using the peqGOLD plasmid Miniprep Kit I (peqLab, Erlangen, Germany) and purified using NucleoSpin Gel and PCR Clean-up (Macherey-Nagel, Düren, Germany) according to the supplier's protocol. Transformation of *E. coli* was performed as described (Sambrook and Russel, 2001). Transformation of *Pseudomonas* strains was performed according to Choi *et al*. (Choi et al., 2006) by means of electroporation (2500 V, Equibio EasyjecT Prima, Kent, UK).

The plasmid pA-EGFP_B_lac (8802 bp) was based on the pA-EGFP_B backbone, which was isolated via PCR using the primers F5 and B6 (Table 2). The 7161 bp fragment was purified via agarose gel-electrophoresis digested with *Xba*I and *Sac*I and ligated with purified and identically digested PCR-fragments using F3 and B4 as primers (1655 bp) and pLac_alaD_ω-TA (encoding the *lac*-regulatory system) as template. pA-EGFP_B_tac (8683 bp) was constructed in analogy to pA-EGFP_B_lac using primers F5 and B6 and pA-EGFP_B as template for the plasmid backbone and primers F1 and B1 and pTAC:LS:AGPPS2 as template for the *tac*-regulatory system (1546 bp). For the construction of plasmid pStyAB_tac (7948 bp), pStyAB served as PCR template using primers F5 and F6. The resulting 6426 bp fragment was digested with *Xba*I and *Sac*I and ligated with a purified and identically digested PCR-fragment (1546 bp) isolated via PCR with the primers F1 and B2 and pTAC:LS:AGPPS2 as template. The constructs were verified by digestion with different restriction enzymes and DNA sequencing.

**Table 2:** **Oligonucleotide primers and probes used in this study**.

**Primer**	**Characteristics**	**Primer sequence (5^′^ → 3^′^)^a^**
F1	LacIq_tac_Fw_*Xba*I	GCGTCTAGAACGCTGCCCGAAATTCCGAC
B2	LacIq_tac_Bw_*Sca*I	CGCAGTACTGTTGGTCAATTGCTCGTGAA
F3	LacI_LacUV5_Fw_*Xba*I	GCGTCTAGAAATTCTCATGTTAGTCATGC
B4	LacI_LacUV5_Bw_*Sca*I	GCGAGTACTGGTTCCTAGATCCTGTGTGA
F5	pCOM_AMP_Fw_*Sac*I	GCGAGTACTGGAGAATTCCATATGAAAAAGC
B6	pCOM_AMP_Bw_*Xba*I	GCGTCTAGAAAATAATCGGCATTAAGTGA
ileS_for	ileS genomic marker	GGACAACCCATACAAGACC
ileS_rev	ileS genomic marker	TCAAAGCACCAGTTCACC
styA_for	styA plasmid marker	GGCTGGTAGAGACGGTAG
styA_rev	styA plasmid marker	CTGAGGAGTTTGGTTATTTCG
**Probes**	**Characteristics**	**Probe sequence (5**′ → **3**′**)**
ileS	ileS genomic marker	FAM-TCCGCGCCCTGGCCGA-BHQ-1
styA	styA plasmid marker	HEX-TGGGAGCCTTGAGATCACCGTAG-BHQ-1

*^a^Restriction sites are underlined*.

### Growth conditions and clonal-variability experiments

Cultivations were carried out in screw-capped, baffled Erlenmeyer shaking flasks at a gas/liquid ratio (v/v) of 9:1, 200 rpm, and 30°C in a Multitron shaker (Infors, Bottmingen, Switzerland). Inoculation and cultivation for clonal variability experiments were carried out as described (Lindmeyer et al., 2015). In short, single colonies (clones) from LB agar plates, which had been incubated for 12–16 h, were picked, grown separately for 6–8 h in liquid LB-precultures, and transferred as a 1% (v/v) inoculum into an overnight M9^*^ preculture, by which the main M9^*^ culture was inoculated to a starting cell concentration of 0.02–0.03 g cell dry weight (CDW) per liter. Recombinant gene expression was induced with 1 mM isopropyl β-D-1-thiogalactopyranoside (IPTG) or 0.025% (v/v) dicyclopropyl ketone (DCPK) 3.5 h after inoculation for P_*lac*UV5_- and P_*tac*_- or P_*alkB*_-based expression vectors, respectively. After induction, incubation was continued for 4 h, before cells were harvested by centrifugation and resuspended to 0.5 g_CDW_ L^−1^ in 50 mM Potassium phosphate (KPi) buffer (pH 7.4) supplemented with 0.5% (w/v) citrate or glucose. Styrene epoxidation assays with nitrogen limited cells (resting-cells) were performed in cell suspension aliquots of 1 mL distributed into 10-mL Teflon sealed Pyrex tubes and incubated for 5 min at 30°C in a rotary shaker (Aquatron, Infors) at 300 rpm. After this equilibration time, the biotransformation was started for 10 min by the addition of 1.5 mM for styrene and stopped by addition of 1 mL ice-cold diethyl ether containing 0.2 mM *n*-decane as internal standard. After 1 min extraction by mixing and phase separation via centrifugation, the organic phase was transferred into a GC-vial for analysis.

For the quantification of variability in clonal-variability experiments, the following equations were used for the determination of the coefficient of variation (*c*_ν_) and the experimental error (σ_*exp*_):

Coefficient of variation:

(1) $$c_{\nu }=\frac{\sigma_{1,2,\ldots ,n-1,n}}{\bar{x_{1,2,...,n-1,n}}}$$

Experimental Error:

(2) $$\begin{array}{l} \sigma_{exp}=\sigma (\frac{x_{1,A}}{{\bar{x}}_{1}},\frac{x_{1,B}}{{\bar{x}}_{1}},\frac{x_{2,A}}{{\bar{x}}_{2}},\frac{x_{2,B}}{{\bar{x}}_{2}},\ldots ,\\ \;\frac{x_{(n-1),A}}{{\bar{x}}_{\left(n-1\right)}},\frac{x_{(n-1),B}}{{\bar{x}}_{\left(n-1\right)}},\frac{x_{n,A}}{{\bar{x}}_{n}},\frac{x_{n,B}}{{\bar{x}}_{n}})\cdot 100\% \end{array}$$

in which σ_1, 2, …, *n*−1, *n*_ is the standard deviation of the specific StyAB activity among the tested colonies, $\bar{x_{1,2,\ldots ,n-1,n}}$ the mean specific StyAB activity among the tested colonies, *n* the amount of tested colonies, $\bar{x_{n}}$ the mean specific activity of one colony, and *x*_*n, A*_ and *x*_*n, B*_ the styrene oxide epoxidation activity of one colony determined in duplicates (*A, B*). The calculation of the coefficient of variation based on specific eGFP fluorescence (defined as eGFP fluorescence intensity normalized to the cell density in terms of OD_450_, see below) was carried out using the same equations replacing specific activity with specific fluorescence. Based on the assumption that, for a consistent biocatalyst performance, the coefficient of variation should not exceed 20%, strains were classified to exhibit low clonal variabilities, if the variation did not exceed 20% (Lindmeyer et al., 2015).

### Analytical procedures

Biomass concentrations were determined by monitoring the optical density at 450 nm using a Libra S11 spectrophotometer (Biochrom Ltd., Cambridge, UK). Correlations of the cell dry weight (CDW) concentration with OD_450_ were reported before and are given in the following per unit OD_450_: *P. putida* DOT-T1E: 0.211 g_CDW_ L^−1^ (Lindmeyer et al., 2015), *P. putida* KT2440: 0.235 g_CDW_ L^−1^ (Lindmeyer et al., 2015), *E. coli* JM101: 0.166 g_CDW_ L^−1^ (Blank et al., 2008a) and *P. taiwanensis* VLB120: 0.186 g_CDW_ L^−1^ (Halan et al., 2011). Styrene and (*S*)-styrene oxide quantification was performed by GC analysis as described (Kuhn et al., 2010b; Volmer et al., 2014).

For the quantification of the extracellular metabolites glucose, gluconate, and citrate, HPLC analysis was performed using a LaChrome Elite HPLC system (Merck Hitachi, Darmstadt, Germany) equipped with a Trentec 308R-Gel.H column (300x3 mm, Trentec Analysetechnik, Gerlingen, Germany) and coupled to UV (λ = 210 nm) and RI detectors. The following analytic conditions were applied: 40°C, 1.0 mL min^−1^ isocratic flow rate, 5 mM H_2_SO_4_ in demineralized water as mobile phase, 20 μL injection volume.

Specific eGFP fluorescence was measured in microtiter plates using an Infinite M200 reader (Tecan, Mannedorf, Switzerland) at 488 nm and 511 nm for excitation and emission, respectively, with a gain factor of 80. Cells were harvested by centrifugation, washed, resuspended to a cell concentration of ~0.1 g_CDW_ L^−1^ in PBS Buffer (145 mM NaCl, 1.8 mM NaH_2_ PO_4_∙H_2_O, 6 mM Na_2_HPO_4_) containing 10% (v/v) glycerol, and stored at −20°C until measurement. Volumes of 200 μL each were transferred to microtiter plates for OD_450_ measurements (Nunc™ Microwell™ 96-well flat transparent, Nunc, Roskilde, Denmark) and eGFP fluorescence determination (Nunc™ Microwell™ 96-well flat black, Nunc, Roskilde, Denmark). The specific fluorescence [OD${}_{450}^{-1}$] was determined as the ratio of the absolute fluorescence and the respective OD_450_ value. SDS-PAGE (Laemmli, 1970) was performed to visualize StyA.

### Sample preparation for flow cytometry and PCN determination

Samples for flow cytometry and PCN determination were processed as described above. After resuspension in PBS, cells were fixed in a 1% (v/w) paraformaldehyde (PFA) solution in PBS-buffer for 30 min at room temperature, washed with PBS, and stored in a cryo-protective solution [10% (v/v) glycerol in PBS] at −20°C until analysis. To investigate possible influences of the fixation agent on eGFP fluorescence and PCN, a control experiment with different concentrations of PFA in *P. putida* DOT-T1E and *P. putida* KT2440 was performed (Figures S2, S3). Fixation influenced PCN determination, as, upon fixation, generally a lower PCN (25–30%) was detected as compared to unfixed cells (Figure S3).

### Flow cytometry and cell sorting

For flow cytometry experiments, fixed cell suspensions were thawed, filtered by a 30 μm CellTrics mesh (Partec-Sysmex, Münster, Germany), and analyzed by a Beckman-Coulter MoFlo cell sorter (Beckman-Coulter, Pasadena, USA) equipped with a 488 nm blue laser (400 mW) for excitation. Forward (FSC) and side scatter (SSC) signals were recorded using a 488/10 nm band pass filter together with a neutral density filter of 2.0, while eGFP fluorescence (FL1) was recorded using a 530/40 nm band pass filter together with a neutral density filter of 0.3. The MoFlo cell sorter was run using the sheath buffer described by Koch et al. (2013) at 2-fold dilution and was calibrated before analysis using fluorescent beads according to Koch et al. (2013) and eGFP-expressing bacterial cells (*P. putida* KT2440) as a biological standard. Fluorescent (eGFP+) and non-fluorescent (eGFP−) populations were manually gated based on FSC and FL1 intensity, with non-induced *P. putida* DOT-T1E (pA-EGFP_B) cells as the eGFP-negative reference (see Figure S9, I–IV, 0 h). Cell samples were generally analyzed at a speed of 3000 cells s^−1^ and sorted into 8-well PCR strips (G003-SF, Kisker Biotech, Steinfurt, Germany) at a lower speed of 200 cells s^−1^ using the CyCLONE robotic tray (Cytomation, Fort Collins, USA) and the most accurate sorting mode (“single cell,” “one drop”). Wells were prefilled with 7 μL deionized H_2_O, 1000 cells per well were sorted resulting in a final volume of 8 μL, and sorted samples were immediately deep-frozen at −20°C until digital PCR analysis. As a no-template-control for digital PCR, 1000 beads (Fluoresbrite Bright Blue Microspheres, Ø = 0.5 μm, Polysciences, Warrington, USA) were sorted instead of cells. Data were recorded using Summit v4.3 (Beckman-Coulter) and detailed analysis was performed using the Bioconductor framework for R (Hahne et al., 2009).

### PCN determination by droplet digital PCR

PCN determination and calculation were carried out as described by Jahn et al. (2014). Briefly, DNA was extracted from sorted cells by heat treatment at 95°C for 20 min as optimized for *P. putida* DOT-T1E (Figure S4), followed by cooling on ice. Droplet Digital PCR (ddPCR) was performed using a duplex reaction setup with simultaneous detection of the chromosomal reference gene *ileS* and the plasmid-located target gene *styA* using a Taqman assay. Primers as well as FAM- and HEX-labeled probes for *ileS* and *styA* are listed in Table 2. A final 20 μL reaction mixture contained 8 μL of template solution, which was completed by adding 12 μL PCR master mix composed of 10 μL 2x ddPCR Supermix (Bio-Rad, Hercules, USA) and 2 μL of a solution containing primers (final concentration of 900 nM) and probes (final concentration of 250 nM for *ileS*, 125 nM for *styA*). The reaction mixture was mixed, briefly centrifuged at 500 × g for 3 s and transferred to DG8 cartridges (Bio-Rad) for droplet generation with the QX100 system (Bio-Rad). The resulting 40 μL of droplet emulsion were pipetted into twin.tec 96-well PCR plates (Eppendorf, Hamburg, Germany), sealed, and subjected to PCR using the following program: 95°C for 10 min, 40 cycles of 94°C for 30 s and 58°C for 60 s, and 98°C for 10 min, with a ramp rate of 2.5°C s^−1^. A Bio-Rad QX100 droplet reader was used for simultaneous detection of FAM and HEX labeled droplets, and PCN was calculated as the ratio of *styA* and *ileS* concentration per well.

## Results

### Characterization of StyA-eGFP fusion

The fusion construct *styA-eGFP* (Jahn et al., 2014) was chosen as a tool to investigate clonal variability regarding recombinant gene expression and a possible connectivity with intra-population variability. In this construct, *styA*, encoding the oxygenase component of the styrene monooxygenase StyAB from *P. taiwanensis* VLB120, is connected to *eGFP* via a flexible linker sequence (GGC GGC GGC GGC GGC GCC) integrated between the C-terminus of StyA and the N-terminus of eGFP (Figure S1). The glycine-rich peptide linker (Gly)_5_Ala thereby functions as a flexible tether minimizing folding interferences between the fluorescence protein and the tagged protein (Tian et al., 2004). As a first step, StyA-eGFP, encoded on the plasmid pA-EGFP_B and expressed under control of the *alk*-regulatory system, was characterized regarding its catalytic styrene epoxidation performance in whole microbial cells (with the specific styrene epoxidation activity given in U g${}_{\text{CDW}}^{-1}$ as readout) by means of resting-cell activity assays (as described in the Materials and Methods section). For this purpose, *P. taiwanensis* VLB120 was used as host strain, as it is the native host strain of StyAB and was found to show a low clonal variability upon heterologous gene expression under control of the *alk*-regulatory system (Lindmeyer et al., 2015). Native *styAB* expressed from an analogous plasmid (pStyAB) served as the control.

**Figure 1:**
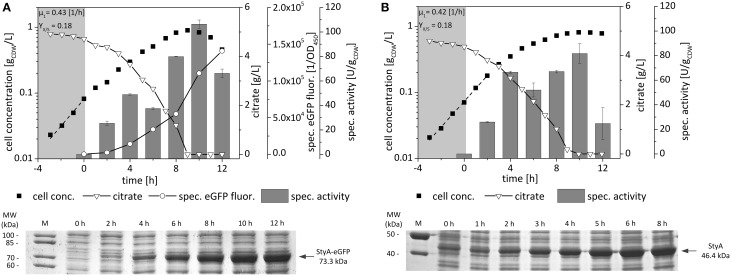
**Growth, expression, and specific styrene epoxidation activity profiles of ***P***. ***taiwanensis*** VLB120 (pA-EGFP_B) (A) and ***P***. ***taiwanensis*** VLB120 (pStyAB) (B) grown on citrate as sole carbon source**. Cells were cultivated in M9* medium and induced with 0.025% (v/v) DCPK after 3 h of growth. Samples for resting-cell activity, specific fluorescence, and SDS-Page analyses were taken at regular time intervals (every 2 h) after induction. For determination of the specific styrene epoxidation activity, cells were harvested, resuspended in KPI buffer containing 0.5% (v/v) citrate, and supplied with 1.5 mM styrene to start the reaction. Growth rates [h^−1^] were determined during exponential phase (dashed line). Yields on citrate were determined after inoculation and at the time point of citrate depletion. Error bars represent standard deviations of two independent assays.

*P. taiwanensis* VLB120 (pA-EGFP_B) and *P. taiwanensis* VLB120 (pStyAB), cultivated in duplicates starting from two individual colonies of each strain, showed a similar behavior in terms of growth rates and yields on citrate (Figure 1). The specific fluorescence of *P. taiwanensis* VLB120 (pA-EGFP_B) cultures correlated well with the steadily increasing specific styrene epoxidation activities and SDS-PAGE band intensities. Compared to native StyAB, for which specific activities of 60–80 U g${}_{\text{CDW}}^{-1}$ were observed for the time range 4–10 h after induction, a higher activity of 106 ± 3.4 U g${}_{\text{CDW}}^{-1}$ was reached with the fusion construct after 10 h of induction. Apparently, expression of the fusion construct was slower as observed by SDS-PAGE band intensities, reaching the maximal level at the beginning of the stationary phase. These results indicate that the *styA-eGFP* expression rate is reduced compared to *styA* gene expression, which might be due to the 5 codon repetitions in the linker sequence leading to a slowdown in translation. The fusion, however, appeared to slightly enhance the maximum *in vivo* oxygenase activity reached after full induction.

To elucidate if the fusion construct also is stable and active, when different expression systems are used, gene expression and specific styrene epoxidation activity of *E. coli* JM101 were studied using three different regulatory systems (*alk, lac, tac*) (Figure S5). Again, specific styrene epoxidation activities maximally achieved with the fusion construct were as high as those obtained with the native StyAB. Thus, the results obtained confirm the applicability of the fusion construct as readout system for recombinant StyAB levels.

### Analysis and quantification of clonal variability by means of fused StyA-eGFP

As next step, fused StyA-eGFP was tested as a tool to analyze the high clonal variability in specific styrene epoxidation activity observed for *P. putida* DOT-T1E (pStyAB). For this purpose, specific fluorescence was measured in 21 individual *P. putida* DOT-T1E (pA-EGFP_B) cultures, originating from individual colonies picked after transformation, and correlated with specific styrene epoxidation activities. For comparison, 8 individual cultures were analyzed of each, *P. taiwanensis* VLB120 showing high StyAB activities (Figure 1) and low respective clonal variability and *P. putida* KT2440 showing low StyAB activities and low clonal variability (Lindmeyer et al., 2015).

**Figure 2:**
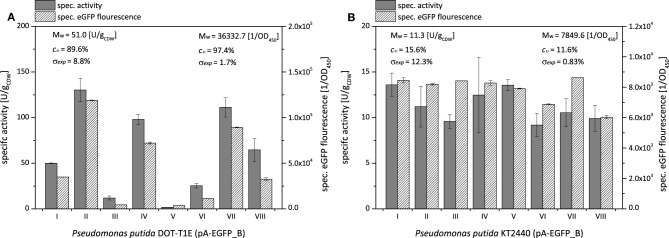
**Clonal variability in specific styrene epoxidation activity and ***styA-eGFP*** expression levels for ***P***. ***putida*** DOT-T1E (A) and ***P***. ***putida*** KT2440 (B) both harboring pA-EGFP_B**. Specific styrene epoxidation activities and expression levels were determined via resting-cell activity assays and specific eGFP fluorescence, respectively. Exemplary results for 8 independent clones are shown. Cultures were induced with 0.025% (v/v) DCPK for 4 h. SDS-PAGE analyses and induction kinetics of respective clones are shown in Figures S6, S7. Average specific styrene epoxidation activities M_*w*_ [U g${}_{\text{CDW}}^{-1}$], average specific fluorescence values [OD${}_{450}^{-1}$], coefficients of variation *c*_ν_ [%], and experimental errors σ_*exp*_ [%] given correspond to 21 and 8 tested cultures of *P. putida* DOT-T1E (pA-EGFP_B) and *P. putida* KT2440 (pA-EGFP_B), respectively.

Using the fusion construct, the high and low specific styrene epoxidation activity variations with *P. putida* DOT-T1E and *P. putida* KT2440 could be confirmed (Figure 2). As it was the case for *P. taiwanensis* VLB120 (Figure 1), specific styrene epoxidation activities of *P. putida* DOT-T1E and *P. putida* KT2440 correlated well with the specific eGFP fluorescence (Figure 2). The average specific styrene epoxidation activities of *P. putida* KT2440 (pA-EGFP_B) (11.3 ± 1.8 U g${}_{\text{CDW}}^{-1}$) were significantly lower as compared to *P. taiwanensis* VLB120 (pA-EGFP_B) and *P. putida* DOT-T1E (pA-EGFP_B) (48.9 ± 10.8 U g${}_{\text{CDW}}^{-1}$ and 51.0 ± 45.7 U g${}_{\text{CDW}}^{-1}$, respectively) and were in accordance with reported activities (Lindmeyer et al., 2015).

**Figure 3:**
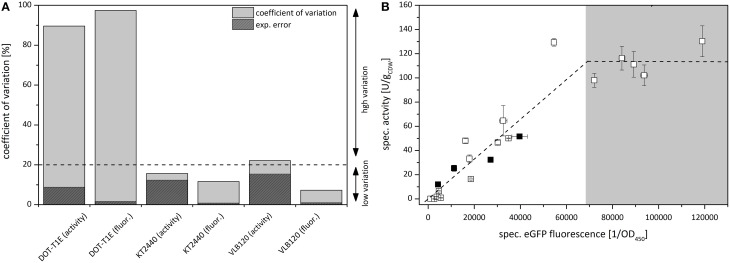
**(A)** Comparison of clonal variability in specific styrene epoxidation activity and specific eGFP fluorescence with *P. putida* DOT-T1E (pA-EGFP_B), *P. putida* KT2440 (pA-EGFP_B), and *P. taiwanensis* VLB120 (pA-EGFP_B) expressing *styA-eGFP* under control of the *alk*-regulatory system. Variations among cultures are displayed via the coefficient of variation *c*_ν_. Dark gray areas represent calculated experimental errors σ_*exp*_ [%]. For *P. putida* KT2440 and *P. taiwanensis* VLB120 harboring pA-EGFP_B, the *c*_ν_ represented by a bar was determined via specific styrene epoxidation activity and specific eGFP fluorescence measurements performed for 8 separate cultures originating from 8 freshly transformed colonies. For *P. putida* DOT-T1E (pA-EGFP_B), *c*_ν_ determination was based on 21 separate cultures. **(B)** Correlation of specific activities of 21 independent *P. putida* DOT-T1E (pA-EGFP_B) cultures with respective specific eGFP fluorescence. The correlation of activity and fluorescence is divided in two sections showing a different interdependence. Black squares represent the 4 colonies which were further analyzed via flow cytometry and digital PCR for PCN determination.

In order to evaluate the fusion construct as a tool to quantify clonal variability, coefficients of variation *c*_ν_ and experimental errors σ_*exp*_ were determined, and respective values obtained via the specific eGFP fluorescence and the specific styrene epoxidation activities 4 h after induction were compared (Figure 3A). With a significantly lower experimental error σ_*exp*_, the determination of the eGFP fluorescence turned out to be more accurate compared to the specific styrene epoxidation activity. This, in consequence, results in a more accurate coefficient of variation *c*_ν_.

The specific eGFP fluorescence of *P. putida* DOT-T1E (pA-EGFPB) cultures correlated linearly with respective specific StyAB activities up to a specific eGFP fluorescence of ~4^*^10^5^ OD${}_{450}^{-1}$ (Figure 3B), indicating that below the corresponding StyA-eGFP expression level, the styrene epoxidation rate is limited by the intracellular oxygenase concentration. At higher expression levels, the specific activity remained constant at around 110 U g${}_{\text{CDW}}^{-1}$ and obviously was not limited by the intracellular StyAB concentration, but rather by substrate availability (i.e., mass transfer limitation for styrene and/or O_2_ and/or a limiting NADH regeneration via glucose metabolism). Thus, regarding the analysis of clonal variability, the fusion construct combined with specific fluorescence determination outperformed specific activity determination not only in terms of accuracy but also with respect to the range of expression levels which can be detected.

### The clonal variability in gene expression observed for *P. putida* DOT-T1E is characterized by a heterogeneous subpopulation distribution

**Figure 4:**
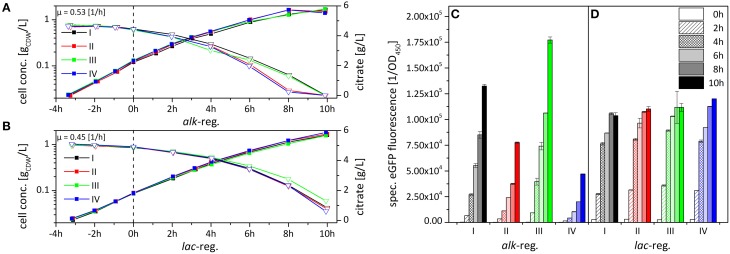
**Comparison of growth behavior (A,B) and variability in StyA-eGFP levels (C,D) upon expression under the control of the ***alk***- (A,C) and the ***lac***- (B,D) regulatory systems in ***P***. ***putida*** DOT-T1E**. Results for 4 representative cultures are shown each for *P. putida* DOT-T1E (pA-EGFP_B) and *P. putida* DOT-T1E (pA-EGFP_B_lac) (see text for details on clone selection). Cells were cultivated in M9^*^ medium supplement with 0.5% (w/v) citrate. Induction was initiated at 0 h by the addition of 0.025% (v/v) DCPK or 1 mM IPTG for the *alk*- or the *lac*-based expression, respectively. The specific fluorescence was determined via eGFP fluorescence measurement and normalization to the cell concentration. Error bars give the standard deviations of two independent fluorescence measurements.

**Figure 5:**
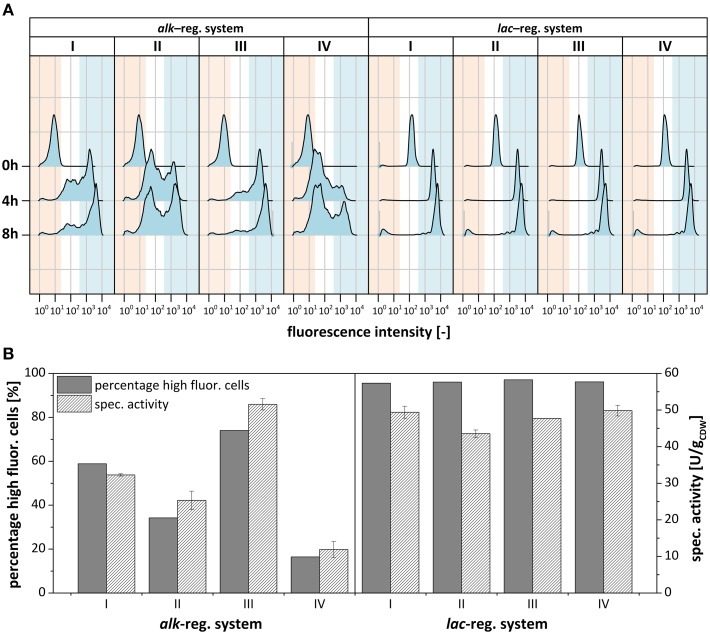
**Subpopulation formation of recombinant ***P. putida*** DOT-T1E regarding ***styA-eGFP*** expression determined via flow cytometry**. **(A)** Flow cytometry analysis of cultures visualized in Figure 4 expressing the fusion construct *styA-eGFP* under control of the *alk*- (left) and the *lac*-regulatory systems (right) before, 4 h after, and 8 h after induction. Populations are split into non-fluorescing (red), low- to medium-fluorescing (white) and high-fluorescing (blue) subpopulations. **(B)** Bar chart correlating the percentage of cells showing high eGFP fluorescence and respective specific styrene epoxidation activities of the whole population after 4 h of induction.

To elucidate if and how the clonal variability observed for *P. putida* DOT-T1E (pA-EGFP_B) depends on subpopulation formation, the subpopulation structure in individual cultures was analyzed via flow cytometry. Since clonal variability did not appear using the *lac*- instead of the *alk*-regulatory system in *P. putida* DOT-T1E (Lindmeyer et al., 2015), pA-EGFP_B_lac was used for comparison. As plasmid backbone and fusion construct were kept identical, emerging differences in gene expression can be attributed to the regulatory system employed. Growth physiology (Figures 4A,B) and *styA-eGFP* expression dynamics (Figures 4C,D) were investigated in detail for 4 cultures each of *P. putida* DOT-T1E, harboring pA-EGFP_B (Figures 4A,C) or pA-EGFP_B_lac (Figures 4B,D) including the single cell level (Figure 5). To distinguish between subpopulations and uncover dynamics in recombinant gene expression, flow cytometry analysis was performed before induction as well as 4 and 8 h after induction.

Only minor variability in growth was observed during cultivation of *P. putida* DOT-T1E (pA-EGFP_B_lac) clones (Figures 4A,B), whereas *P. putida* DOT-T1E (pA-EGFP_B) cultures showed higher growth variation after induction with a negative correlation of the average eGFP-fluorescence and the overall growth rate. This can be explained by stress induced by *styA-eGFP* overexpression (Figures 4A,C), as it has been observed for *styAB* overexpression in *E. coli* (Bühler et al., 2008).

In contrast to the high clonal variability in StyA-eGFP content among the 4 chosen *P. putida* DOT-T1E (pA-EGFP_B) cultures (Figure 4C), *P. putida* DOT-T1E (pA-EGFP_B_lac) cultures did not vary regarding StyA-eGFP content and respective expression dynamics (Figure 4D).

These clonal cell populations were further investigated by flow cytometry (Figure 5). Before induction, minor leaky gene expression was observed with the *lac*-regulatory system, whereas no leaky expression was detected with the *alk*-regulatory system (Figures 4D, 5A). For cells harboring the *lac*-vector, mainly one population with high fluorescence intensity and thus low intra-population variability was found after 4 h of induction meaning that cells homogenously expressed *styA-eGFP* at a high-level. After 8 h of induction, when the growth rate decreased, a non-fluorescing subpopulation had increased in size. Such a small non-fluorescing subpopulation (< 10%) also developed upon *alk*-based expression. In stark contrast to this observation, *P. putida* KT2440 (pA-EGFP_B_lac) as well as *P. putida* KT2440 (pA-EGFP_B) showed the formation of large non-fluorescing subpopulations, which, under comparable growth conditions, were very similar in size (63 and 62% of all cells, respectively; Figure S2B). This indicates that *P. putida* KT2440, in contrast to *P. putida* DOT-T1E, forms large fluorescence-free subpopulations upon both *alk-* and the *lac*-based expression despite the presence of kanamycin. In *P. putida* KT2440, such fluorescence loss was related to plasmid loss (Jahn et al., 2014).

As it was the case for *P. putida* DOT-T1E (pA-EGFP_B_lac), almost all cells in all 4 clonal *P. putida* DOT-T1E (pA-EGFP_B) cultures actively expressed the eGFP construct. However, in contrast to *P. putida* DOT-T1E (pA-EGFP_B_lac), *P. putida* DOT-T1E (pA-EGFP_B) cultures developed, beside a small non-fluorescing subpopulation, at least two distinct subpopulations with different recombinant protein contents (high and low) reflecting high intra-population variability (Figure 5A). Considering the different abundancies of highly fluorescing cells among individual cultures of *P. putida* DOT-T1E (pA-EGFP_B), average eGFP fluorescence variations (Figure 4C) correlated well with the respective distribution of the two subpopulations (Figure 5A). It can be concluded that clonal variability in specific styrene epoxidation activity depends on the sizes of the two subpopulations with high or low oxygenase expression levels: the larger the size of the high fluorescing subpopulation the higher the mean specific StyAB activity of the total population (Figure 5B).

### Variation in PCN is not the cause for the intra-population and clonal variabilities observed for *alk*-based gene expression in *P. putida* DOT-T1E

**Figure 6:**
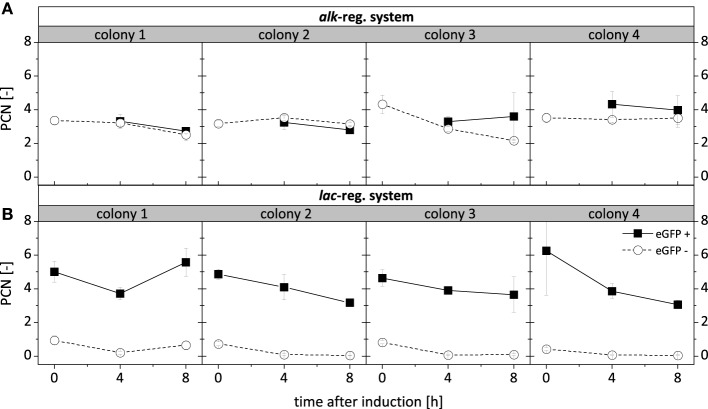
**Average PCN per cell after 0 h, 4 h and 8 h of induction of ***P. putida*** DOT-T1E recombinants**. One thousand cells originating from the 8 respective cultures visualized in Figure 5 expressing the fusion construct *styA-eGFP* under control of the *alk*- **(A)** and the *lac*- **(B)** regulatory systems were sorted according to defined gates (Figure S9) for fluorescing (eGFP+) and non-fluorescing (eGFP−) cells. PCNs were determined in quadruplicates for each subpopulation and are presented as an average value with standard deviations.

The intra-population variability in recombinant gene expression observed for *P. putida* DOT-T1E (pA-EGFP_B) (Figure 5A) can be caused by interactions of expression regulation and the host specific regulatory network or by PCN variation, which might occur due to an interference of the *alk*-regulatory system with plasmid replication/segregation. In order to test this, cells of different subpopulations were sorted and the PCN was determined in respective high-fluorescing (eGFP+) and non-fluorescing (eGFP−) cells of both *P. putida* DOT-T1E (pA-EGFP_B) and *P. putida* DOT-T1E (pA-EGFP_B_lac) (Figure 6). For *P. putida* DOT-T1E (pA-EGFP_B), the average PCN per cell (3-4) was the same for eGFP+ and eGFP− cells and thus did not correlate with the respective recombinant StyA-eGFP fluorescence. A difference in PCN was found for eGFP+ (PCN = 3-6) and eGFP− (PCN = 1) cells of *P. putida* DOT-T1E (pA-EGFP_B_lac). Thus, the appearance of the small non-fluorescing subpopulation of *P. putida* DOT-T1E (pA-EGFP_B_lac) obviously is associated with instable plasmid proliferation. However, the expression variability exhibited by *P. putida* DOT-T1E (pA-EGFP_B) was not related to plasmid proliferation, indicating that interactions of the *alk*-regulatory system and the host specific regulatory network finally lead to the observed high clonal variability in expression and biocatalytic activity.

## Discussion

The occurrence of two distinct cell states in an isogenic bacterial culture is a classical phenomenon of intra-population variability, with both cells not acting as catalysts although being alive and cells being highly productive (Müller et al., 2010). For the elucidation of such a binary mechanism in bioprocesses, flow cytometry can provide quantitative measures on cell state variations (Diaz et al., 2010). In this study, flow cytometry analysis of *P. putida* DOT-T1E (pA-EGFP_B) cultures has revealed the formation of two main subpopulations (high and low *styA-eGFP* expression levels) varying in size in individual cultures originating from different isogenic colonies. This variability in subpopulation sizes was found to be the cause for the observed clonal variability in biocatalytic performance (in terms of specific styrene epoxidation activity). Furthermore, the observed intra-population variability seems to be caused by interactions of the *alk*-regulatory system and the host specific regulatory network. This situation differed for expression in *P. putida* KT2440, for which a regulatory system-independent intra-population variability, but no clonal variability has been found (Table 3; Jahn et al., 2012; Lindmeyer et al., 2015). A main difference between *P. putida* KT2440 and *P. putida* DOT-T1E lies in the tolerance against organic solvents. In contrast to solvent-sensitive *P. putida* KT2440, *P. putida* DOT-T1E harbors the self-transmissible megaplasmid pGRT1 (133 kbp) encoding genes involved in solvent tolerance such as the most relevant solvent efflux pump TtgGHI. However, on the mechanistic level, solvent tolerance and variability do not seem to be interconnected (Lindmeyer et al., 2015). These observations emphasize the diversity among different *P. putida* strains. In the following, the utility of eGFP-fusion as a tool to investigate intra-population variability, the connectivity of clonal variability and subpopulation formation, and implications of the results obtained in this study for biocatalyst and bioprocess engineering are discussed.

**Table 3:** **Expression variabilities observed for ***P. putida*** DOT-T1E and ***P. putida*** KT2440**.

**Type of expression variability**	***P. putida*** **DOT-T1E**		***P. putida*** **KT2440**	
	***lac*-based**	***alk*-based**	***lac*-based**	***alk*-based**
Intra-population	LOW^a^	HIGH^a^	HIGH^a^	HIGH^a, c^
Clonal	LOW^a^	HIGH^a^	LOW^b^	LOW^b^

*^a^This study; ^b^Lindmeyer et al. (2015); ^c^Jahn et al. (2012)*.

### Fusion to *eGFP* as tool to analyze clonal variability and its connectivity to intra-population variability

Transcriptional fusion of GFP or its derivatives (Shaner et al., 2005) to target proteins is a commonly used non-invasive technique to assess recombinant gene expression by flow cytometry (Tracy et al., 2010). Such biosensors are applied to analyze intra-population variability and/or to quantify/optimize recombinant protein production (Burmølle et al., 2005; Sevastsyanovich et al., 2009). Due to its reliability and simplicity, the *styA-eGFP* fusion construct was shown to enable a highly accurate calculation of the coefficient of variation as a measure for clonal variability. Thereby, the detectable range of expression levels was found not to be biased by substrate availability as it was the case for the specific activity as readout (Figure 3).

In all strains tested, the fusion construct showed a high and even superior styrene epoxidation performance compared to native StyAB. Obviously, *in vivo* styrene epoxidation activity was not negatively affected by the fusion of StyA to eGFP or the linker in-between. Since protein linkers have effects on the folding stability, oligomeric state, proteolytic resistance, and solubility of fusion proteins (Robinson and Sauer, 1998; Xue et al., 2004), one may speculate that such effects positively influenced the *in vivo* styrene epoxidation performance of StyA-eGFP StyB.

For *P. putida* DOT-T1E containing the fusion construct, the variable specific styrene epoxidation activity among cultures originating from different isogenic colonies correlated linearly with the specific eGFP fluorescence up to a threshold level of around 110 U g${}_{\text{CDW}}^{-1}$, which was not exceeded at further increasing fluorescence intensities, i.e., expression levels (Figure 3B). This may be explained by substrate limitation at high expression levels. Considering the three StyAB substrates styrene, O_2_, and NADH, limitation by the former two can be excluded since cell densities used in resting-cell assays were optimized to avoid respective substrate depletion, as it was reflected by constant activities over 20 min (data not shown) (Panke et al., 1998; Lindmeyer et al., 2015). This points to a competition of the increasing epoxidation-related NADH demand with other intracellular NADH dependent reactions, especially respiration, and/or stress imposed by oxygenase overexpression affecting the metabolic capacity (Bühler et al., 2006, 2008; Blank et al., 2008b).

### The connectivity of clonal variability and subpopulation formation

Subpopulation formation has already been reported for *P. putida* KT2440 and was related to variable DNA contents of cells in chemostat cultures or bistability during recombinant gene expression (Figure S2B) (Jahn et al., 2012; Lieder et al., 2014). In contrast to *P. putida* KT2440 (pA-EGFP_B), which showed the formation of a large non-fluorescing subpopulation associated with plasmid loss (Jahn et al., 2014), the PCN was found not to be reduced in eGFP− cells of *P. putida* DOT-T1E (pA-EGFP_B), indicating that no or low styrene monooxygenase production cannot be ascribed to the lack of the respective genetic information and plasmid replication/segregation. This together with the finding that the clonal variability observed for *P. putida* DOT-T1E was found not to depend on the type of heterologous enzyme, antibiotic resistance mechanisms, nor on inducer toxicity (Lindmeyer et al., 2015) indicates that the observed clonal and intra-population variabilities in recombinant gene expression are related to interactions of the *alk*-regulatory system with the host regulatory network. A similar behavior can be assumed for *P. putida* S12, for which a very similar clonal variability for recombinant gene expression under control of the *alk-*regulatory system has been found (Lindmeyer et al., 2015).

The *alk*-regulatory system, which originates from *P. putida* GPo1 (van Beilen et al., 1994; Staijen et al., 1999), has been intensively investigated and employed as powerful tool for recombinant gene expression in *E. coli* and *Pseudomonas* strains (Yuste et al., 1998; Canosa et al., 1999, 2000; Panke et al., 2007). It features a positive feedback mechanism governed by the regulator AlkS. Regarding *alkS* expression, a constitutive promoter (P_*alkS*1_) establishes a basic AlkS level, whereas, in the presence of an inducer such as alkanes or DCPK, AlkS activates its own formation from a second promoter (P_*alkS*2_) and gene expression at the P_*alkB*_ promotor. In *Pseudomonas* strains, the *alk*-regulatory system is subject to catabolite repression, e.g., by glucose, but not by citrate, pyruvate, and glycerol, whereas catabolite repression is absent in *E. coli* (Staijen et al., 1999; Yuste and Rojo, 2001; Dinamarca et al., 2002). AlkS belongs to the unique group of the **L**arge **A**TP-binding regulators of the **L**uxR family (LAL-regulators) (De Schrijver and De Mot, 1999), characterized by their unusually large size (>800 aa) and the presence of an N-terminal ATP/GTP-binding domain and a C-terminal LuxR-like DNA-binding domain. Typically, LAL-regulators act globally controlling various cellular processes via a complex modulation involving the interaction with multiple effectors. They participate in the regulation of diverse types of metabolism in both Gram-negative and Gram-positive bacteria and are proposed to play a role in higher steps of regulatory cascades (Zhao et al., 2010; Guerra et al., 2012). Autoinduction, as described for AlkS, is not a common feature of LAL-regulator proteins (Boos and Shuman, 1998). However, a positive feedback mechanism was proposed by Gerrites et al. for the LAL-regulator protein OrfV from *Pseudomonas alcaligenes* (Gerritse et al., 1998). In a recent publication, the inherent clonal variability of *P. putida* DOT-T1E was proposed to rely on phenotypic predispositions of the source-clone (colony), thereby assuming that concentrations of regulatory molecules, such as AlkS or related compounds, differ from source-clone to source-clone and might be bequeathed to the daughter cells (Lindmeyer et al., 2015). This stochasticity in predispositions of the regulatory network was proposed to affect AlkS/P_*alkB*_ mediated expression. It can be speculated that a certain threshold concentration of AlkS, established via the P_*alkS*1_, must be present before induction to efficiently trigger its own formation upon induction via P_*alkS*2_. After induction of *P. putida* DOT-T1E (pA-EGFP_B) cultures, almost all of these cells expressed the *styA-eGFP* gene at least at a low level. In such a scenario, the AlkS threshold concentration for P_*alkB*_ promotor activation would be lower than that required to trigger its own formation via P_*alkS*2_. Thus, the high intra-population variability observed for *P. putida* DOT-T1E may be attributable to the architecture of the *alk*-regulatory system. In general, depending on the sensitivity of autoinduction and the concentration of the transcription factor (TF), a simple positive feedback regulation motif (TF enhances its own production in the presence of an inducer) leads to a broad distribution in gene expression and, in case of strong autoinduction, can result in a bi-modal behavior under the premise that in some cells the TF concentration is low and in others high (Alon, 2007; Silva-Rocha and de Lorenzo, 2010). In contrast, negative regulation and especially feedback regulation are classified as the most “noise-attenuating” regulatory network motifs and generally provide increased stability and homogenous gene expression regardless of inherent extrinsic and intrinsic noises (Patnaik, 2006). The *lac*-regulatory system applied in this study is not subject to autoregulation, but is under both negative and positive control. Thereby, the low intra-population variability observed may well be attributable to the negative control by LacI.

It is known that, in fully induced cells, the level of recombinant gene expression via P_*alkB*_ is limited by the intracellular AlkS level (Yuste and Rojo, 2001). Thus, variability in intracellular AlkS concentrations would also affect gene expression at the P_*alkB*_ promotor. Variability may further be promoted by the fact that AlkS is a highly instable protein (Yuste and Rojo, 2001; Moreno et al., 2007). *P. taiwanensis* VLB120, *E. coli* JM101, and *P. putida* KT2440 did not show any clonal variability, although bimodality and thus intra-population variability was observed for both *alk-* as well as *lac*-controlled *styA-eGFP* expression in *P. putida* KT2440 (Figure S2B). However, clonal variability characterized by variable subpopulation sizes obviously did not appear in *P. putida* KT2440 (pA-EGFP_B) (Lindmeyer et al., 2015), where intra-population variability was associated with the appearance of a plasmid-free subpopulation (Jahn et al., 2014). Thus, a possible variability in AlkS levels obviously did not occur or cause expression variability in the three reference strains. In general, the clonal consistency of StyAB-expression levels and activities in these strains indicates that certain stochastic variabilities (“noise”) may either be reduced or not interact with the *alk*-regulatory system.

Overall, the observed clonal variability in StyA-eGFP levels might be the result of heterogeneous intracellular concentrations of AlkS and/or other regulatory compounds and may be promoted by AlkS features such as its instability, its autoinduction, and its connectivity to the regulatory network of *Pseudomonas* strains. Further, studies are in progress to investigate such connectivity with intracellular AlkS concentrations with a special emphasis on constitutive *alkS* overexpression.

### Implications for biocatalyst and bioprocess engineering

Recombinant *P. putida* DOT-T1E harboring the fusion-construct under control of the *lac*-regulatory system showed stable and reproducible gene expression levels auguring well for application. Before induction, specific eGFP fluorescence measurements revealed a low signal, indicating a slight leakiness of this expression system (Figure 5). For the P_*lac*UV5_–*lac*I system, such leaky expression also was reported in *E. coli* (Baneyx, 1999; Zhou et al., 2004). After induction, the high *styA-eGFP* expression levels were accompanied by growth inhibition owing to an increased stress caused by oxygenase overproduction as it also was observed for *E. coli* (Bühler et al., 2008; Leak et al., 2009). Beyond that, specific styrene epoxidation activities of *P. putida* DOT-T1E (pA-EGFP_B_lac) decreased after extended induction periods by 67% (8 h, Figure S8), which also can be explained by increasing oxygenase-related stress and/or by inclusion body formation (Yanase et al., 2002; Fahnert et al., 2004), aggregation of misfolded proteins caused by aminoglycoside antibiotics (Ling et al., 2012; Goltermann et al., 2013), or the formation of subpopulations not contributing to the biotransformation. However, the increase in population-wide specific eGFP fluorescence between 4 and 8 h after induction indicates that the development of the small subpopulation lacking the fusion protein (~10% of the cells) did not decisively contribute to the reduced styrene epoxidation performance.

Instable proliferation of the plasmid pA-EGFP_B has been observed for *P. putida* KT2440 (Jahn et al., 2014), which appeared to develop a mutation- and plasmid-independent adaptive resistance to kanamycin leading to the formation of a large plasmid-free inactive subpopulation. This subpopulation formation was independent of the regulatory system employed as very similar subpopulation sizes upon *alk-* and *lac*-controlled *styA-eGFP* expression in *P. putida* KT2440 were detected under comparable conditions (Figure S2B). Strain-inherent efflux pumps have been reported to be involved in such adaptive antibiotic resistance (Sánchez-Romero and Casadesús, 2014). However, the small size of the plasmid-free population of *P. putida* DOT-T1E (pA-EGFP_B_lac) and the time point of its formation, both in contrast to the behavior of *P. putida* KT2440 (Jahn et al., 2014), indicate that a substantial adaptive kanamycin resistance did not develop.

Although, only 3–5 plasmid copies per cell were detected in recombinant *P. putida* DOT-T1E, high expression levels and styrene epoxidation activities up to ~130 U g${}_{\text{CDW}}^{-1}$ (Figure 2) were achieved, exceeding those of *E. coli* (up to 100 U g${}_{\text{CDW}}^{-1}$) exhibiting PCNs up to 100 per cell (Jahn, unpublished data). The high gene expression levels achieved from a low gene copy number, as it also has been described for other *Pseudomonas* strains (Panke et al., 1999), can be considered beneficial for the construction of stable production strains, given that reproducibility issues as described in this study can be avoided. Unequal partitioning of low PCNs (< 3) might cause intra-population variability. Such heterogeneity can be overcome via the use of toxin-antitoxin systems or systems actively controlling equal segregation (Jahn et al., 2015). Alternatively, chromosomal integration can be combined with the careful selection of a tunable expression system, thereby avoiding difficulties related to antibiotic markers, plasmid instability, gene overexpression, and a metabolic burden by high DNA load (Birnbaum and Bailey, 1991; Andersson and Hughes, 2010). Considering the variability issues, which arose using the *Pseudomonas*-derived *alk*-regulatory system, the chosen regulatory system should be as orthologous as possible to the host regulatory network to avoid interactions with host specific sigma factors (Canosa et al., 1999), small regulatory RNAs (Moreno et al., 2012), or carbon catabolite repression (Rojo, 2010). In this sense, the combination of well-chosen tunable regulatory systems and stable optimized *Pseudomonas* chassis strains (Martínez-Garcia et al., 2014; Lieder et al., 2015) augurs well for industrial application with minimal clonal and intra-population variability.

## Conclusion

To the best of our knowledge, the observed intra-population variability displayed by heterogeneous subpopulation sizes in individual cultures is reported for the first time as an underlying principle for clonal variability. Furthermore, the presented results show that the detected variations in recombinant gene expression (i.e., *alk-*based *styA-eGFP* expression in *P. putida* DOT-T1E) are not owing to plasmid instability and PCN but rather depend on the regulatory background of the host strain itself and its interaction with the regulatory system employed. In general, clonal variability and expression-related stress are important factors to be considered for the development of production strains and the exploitation of the beneficial characteristics of *Pseudomonads* such as solvent tolerance, high metabolic capacity, and low gene dosage requirement. Careful selection of tunable expression systems and chromosome-based expression are considered crucial.

### Conflict of interest statement

The authors declare that the research was conducted in the absence of any commercial or financial relationships that could be construed as a potential conflict of interest.
