# Supplementary material for: Variability in subpopulation formation propagates into biocatalytic variability of engineered Pseudomonas putida strains
Source: Front Microbiol. 2015 Oct 1;6:1042. doi: 10.3389/fmicb.2015.01042 (PMC4589675; doi:10.3389/fmicb.2015.01042)
Supplement: Supplementary file 1 [file DataSheet1.PDF]

Supplemental Material

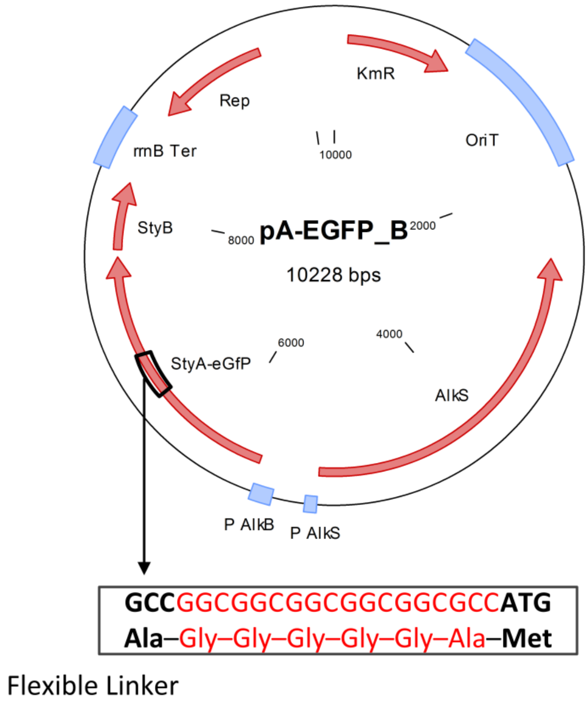

Figure S1: Plasmid map of pA-EGFP_B encoding the fusion construct StyA-eGFP. pA-EGFP_B is a pCOM10 derived vector (AJ302087) harboring the genes encoding the styrene monooxygenase *styA* (AGZ38048.1) and *styB* (AGZ38049.1) of *P. taiwanensis* VLB120 (CP003962). The *styA* gene is connected via a flexible linker $(Gly)_5$Ala to the enhanced version of *gfp* originating from the jellyfish *Aequorea victoria*.

Validation of the fixation procedure

PFA fixation was tested in 4 different strains all harboring plasmids encoding the fusion protein StyA-eGFP, either under control of the *lac*- or the *alk*-regulatory systems: *P. putida* DOT-T1E (pA-EGFP_B_lac), *P. putida* DOT-T1E (pA-EGFP_B), *P. putida* KT2440 (pA-EGFP_B_lac) and *P. putida* KT2440 (pA-EGFP_B). Cultivation was carried out in M9* media supplemented with glucose as sole carbon source. Cells were harvested 4 h after induction and incubated for 30 min at room temperature in either 1% PFA, 4% PFA, or phosphate buffered saline (PBS) as

reference. After washing the cells with PBS, cells were stored in PBS containing 15% glycerol or in 70% EtOH at -20 °C until analysis, which included specific fluorescence determination, flow cytometry analysis, and PCN determination.

Fluorescence analysis revealed that the cross-linking agent PFA used for fixation caused only minor differences in the specific fluorescence detected (Figure S2A). Furthermore, no changes in the ratio of fluorescing and non-fluorescing cells could be determined in fixed and unfixed cells (Figure S2B). Thus, the tested cell fixation procedures do not have a significant influence on eGFP-fluorescence enabling qualitative and quantitative measurements. However, fixation influenced PCN determination, as, upon fixation, generally a lower PCN (25-30%) was detected as compared to unfixed cells (Figure S3).

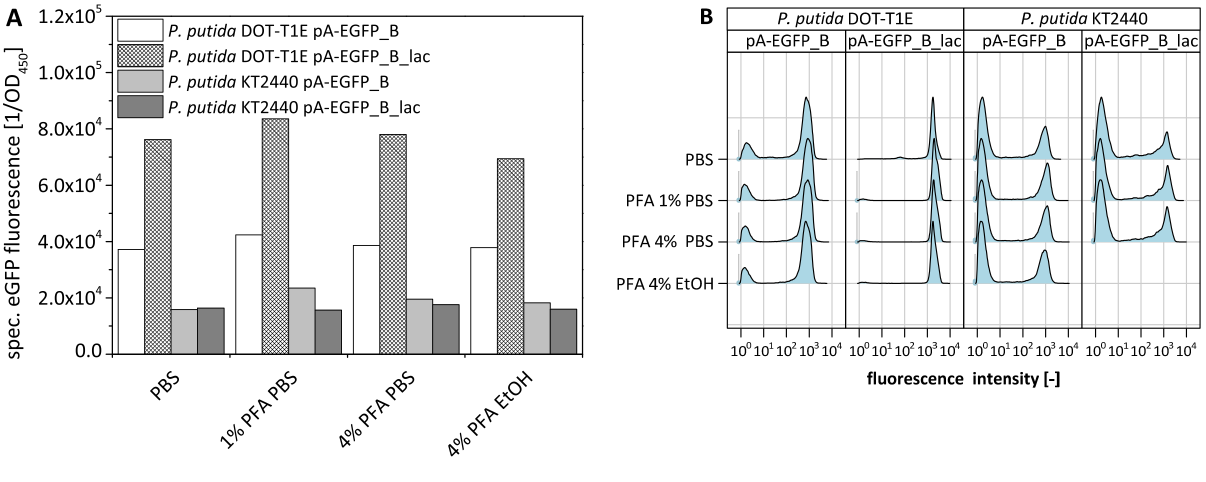

Figure S2: (A) Specific fluorescence intensities of *P. putida* DOT-T1E (pA-EGFP_B), *P. putida* DOT-T1E (pA-EGFP_B_lac), *P. putida* KT2440 (pA-EGFP_B), and *P. putida* KT2440 (pA-EGFP_B_lac) cells determined using a Tecan multiplate reader 4 h after induction. Before analysis, cells were treated with different concentrations of the fixation agent PFA and stored at -20 °C either in PBS buffer supplemented with 15% glycerol (PBS) or in 70% ethanol (EtOH). **(B)** Histograms of the same cultures obtained via flow cytometry. Per measurement, 50,000 cells were analyzed and visualized on a log scale representing the intracellular StyA-eGFP content.

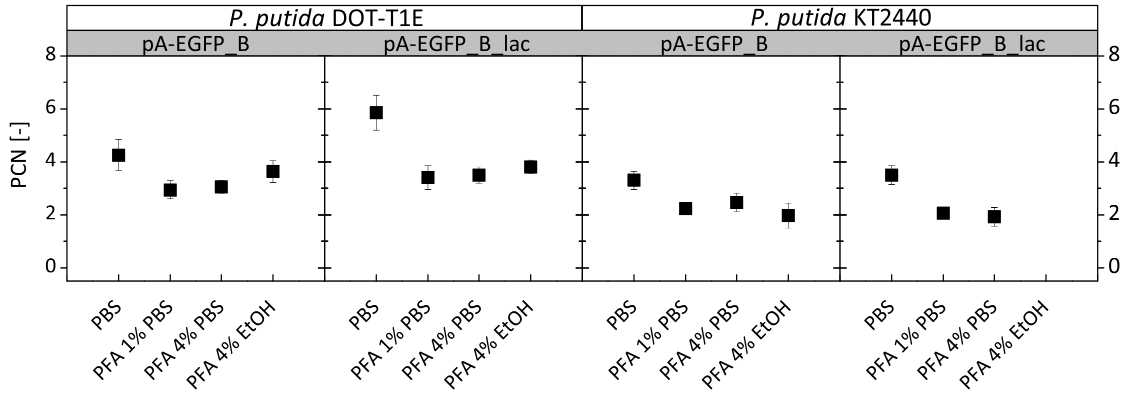

Figure S3: Plasmid copy numbers (PCNs) in *P. putida* DOT-T1E pA-EGFP_B, *P. putida* DOT-T1E (pA-EGFP_B_lac), *P. putida* KT2440 (pA-EGFP_B), and *P. putida* KT2440 (pA-EGFP_B_lac) cells determined 4 h after induction. From each of the 4 cultures introduced in Figure S2 (expressing *styA-eGFP* under control of the *alk*- or the *lac*-regulatory system), 1000 eGFP+ cells were sorted after differential fixation and PCN was determined via Droplet Digital PCR in quadruplicates presented as an average value with standard deviations.

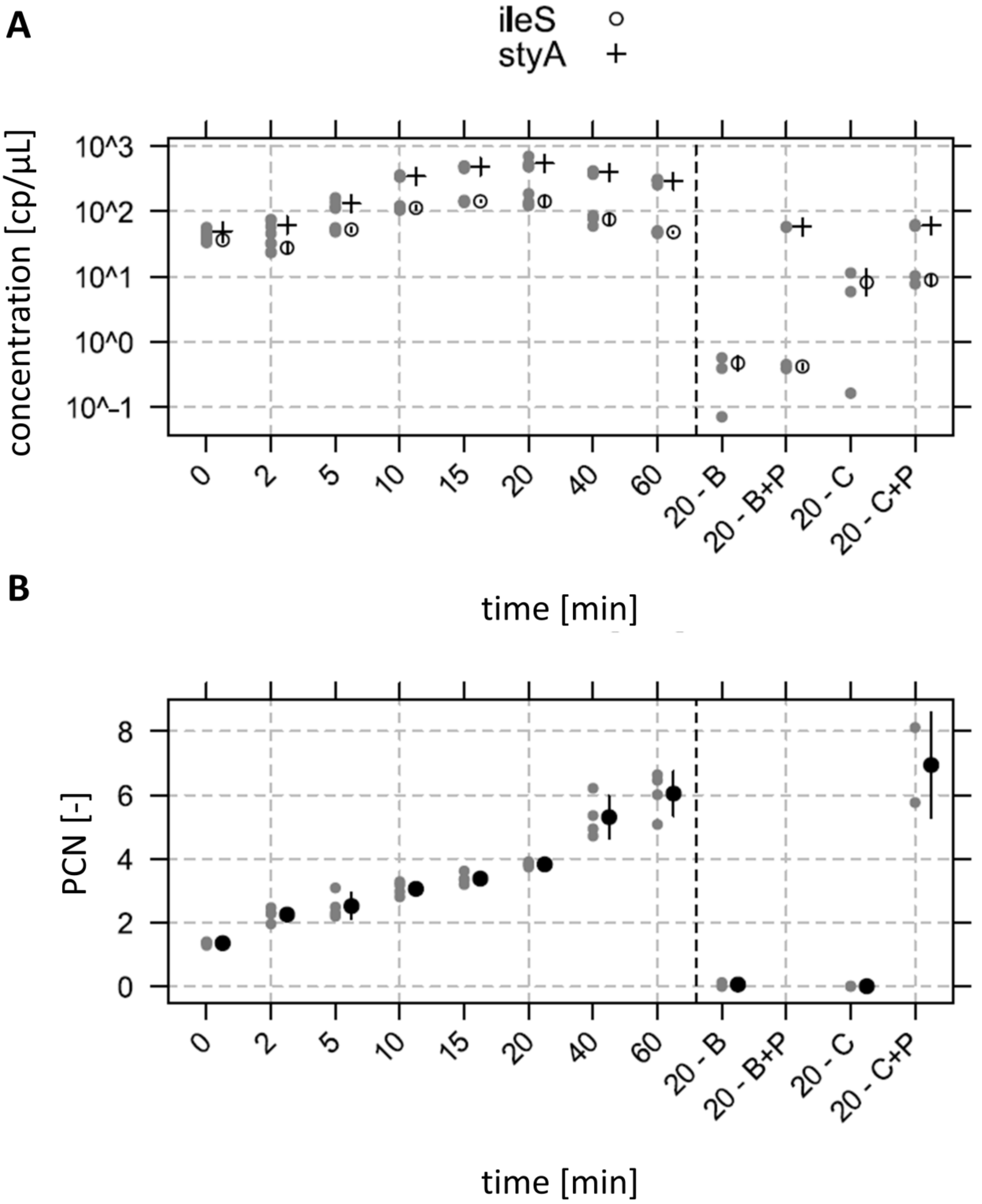

Figure S4: DNA extraction by heating (95 °C) of *P. putida* DOT-T1E pA-EGFP_B_lac cells. Digital droplet PCR for determination of the plasmid copy number (PCN) was performed after heating for different time periods (0−60 min). The template comprised 1000 sorted cells of *P. putida* DOT-T1E pA-EGFP_B_lac after 4 h of induction. PCR was performed with primers targeting a plasmid maker (*styA*) as well as a genomic DNA marker (*ileS*). Panels A and B show respective DNA concentrations (cp/µL, copies/µL) and the PCN calculated as the ratio of *styA* and *ileS* copy numbers. Gray symbols: replicate values. Black symbols: mean ± standard deviation. Control experiments with beads as negative control (B), beads with pipetted plasmid (B+P), plasmid-free cells of *P. putida* KT2440 (C) and plasmid-free cells of *P. putida* KT2440 with pipetted plasmid (C+P) were heated for 20 min.

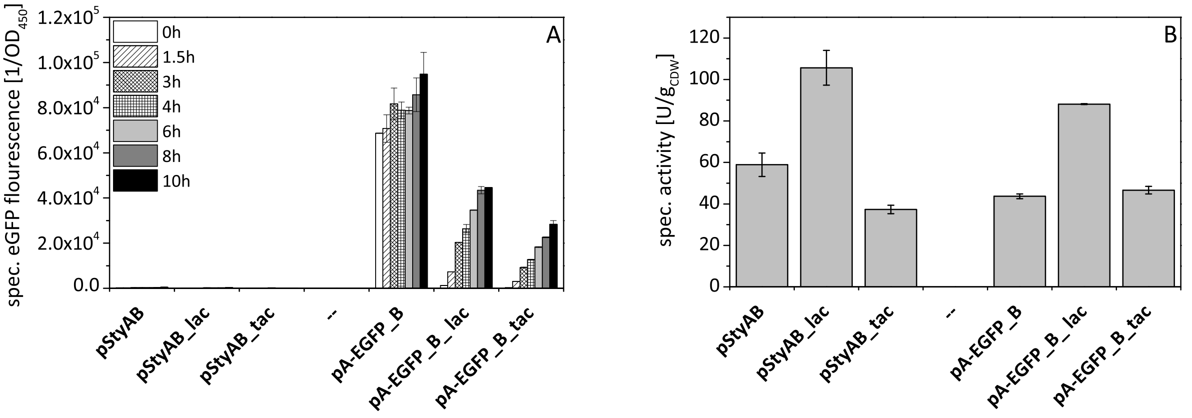

Figure S5: Specific fluorescence **(A)** and specific styrene epoxidation activities **(B)** of *E. coli* JM101 harboring plasmids encoding the styrene monooxygenase StyAB (pStyAB, pStyAB_lac, pStyAB_tac) or the fusion construct StyA-eGFP (pA-EGFP_B, pA-EGFP_B_lac, pA-EGFP_B_tac) under the control of the *alk*-, *lac*- and *tac*-regulatory systems. The specific fluorescence of each induced culture used for activity assays corresponds to the respective StyA-eGFP content after induction with 1 mM IPTG or 0.025% (v/v) DCPK. Specific styrene epoxidation activities were determined via resting-cell assays after 4 h of induction.

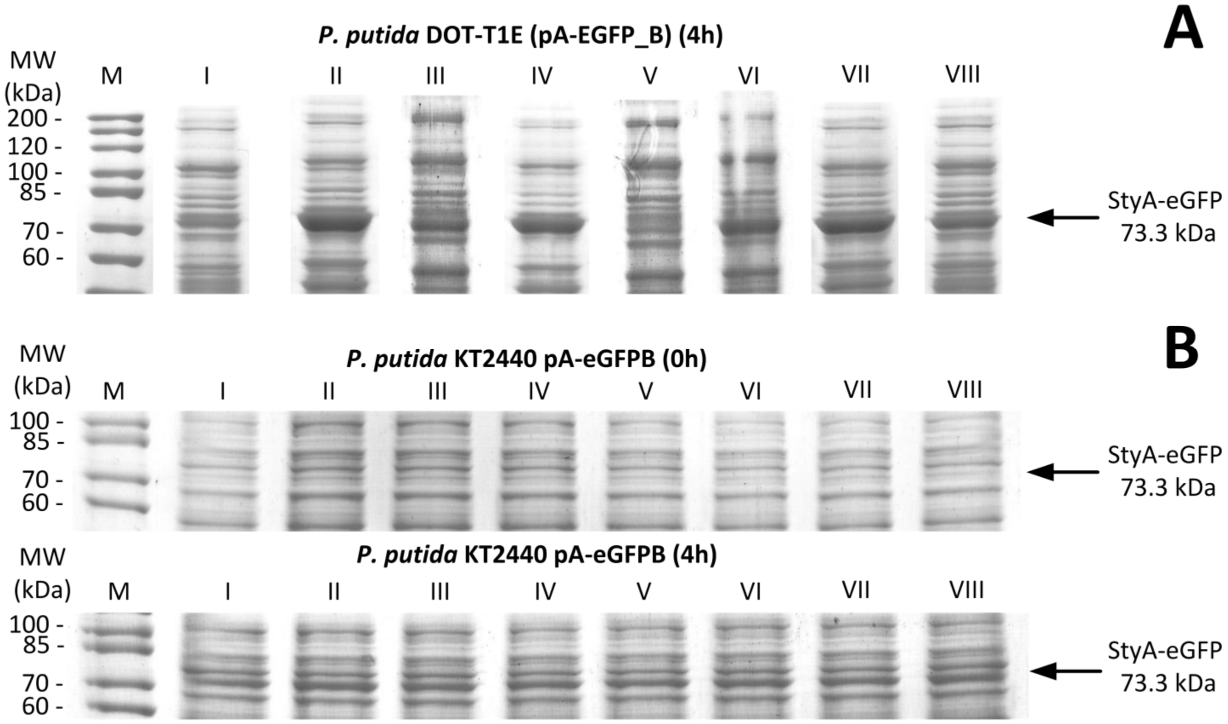

Figure S6: SDS-PAGE analysis of the 8 *P. putida* DOT-T1E (pA-EGFP_B) cultures **(A)** and the 8 *P. putida* KT2440 (pA-EGFP_B) cultures **(B)** described in Figure 2 after 4 h of induction and after 0 and 4 h of induction, respectively.

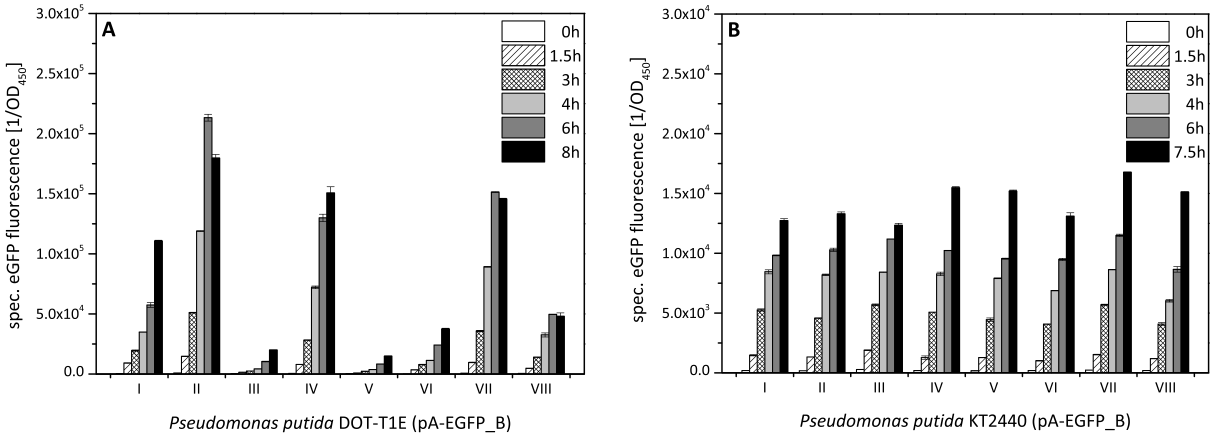

Figure S7: Clonal variability in StyA-eGFP expression levels for *P. putida* DOT-T1E (**A**) and *P. putida* KT2440 (**B**) both harboring pA-EGFP_B determined via specific eGFP fluorescence. Exemplary results for 8 independent clones described in Figure 2 are shown. Cultures were induced with 0.025% (v/v) DCPK for 8 h.

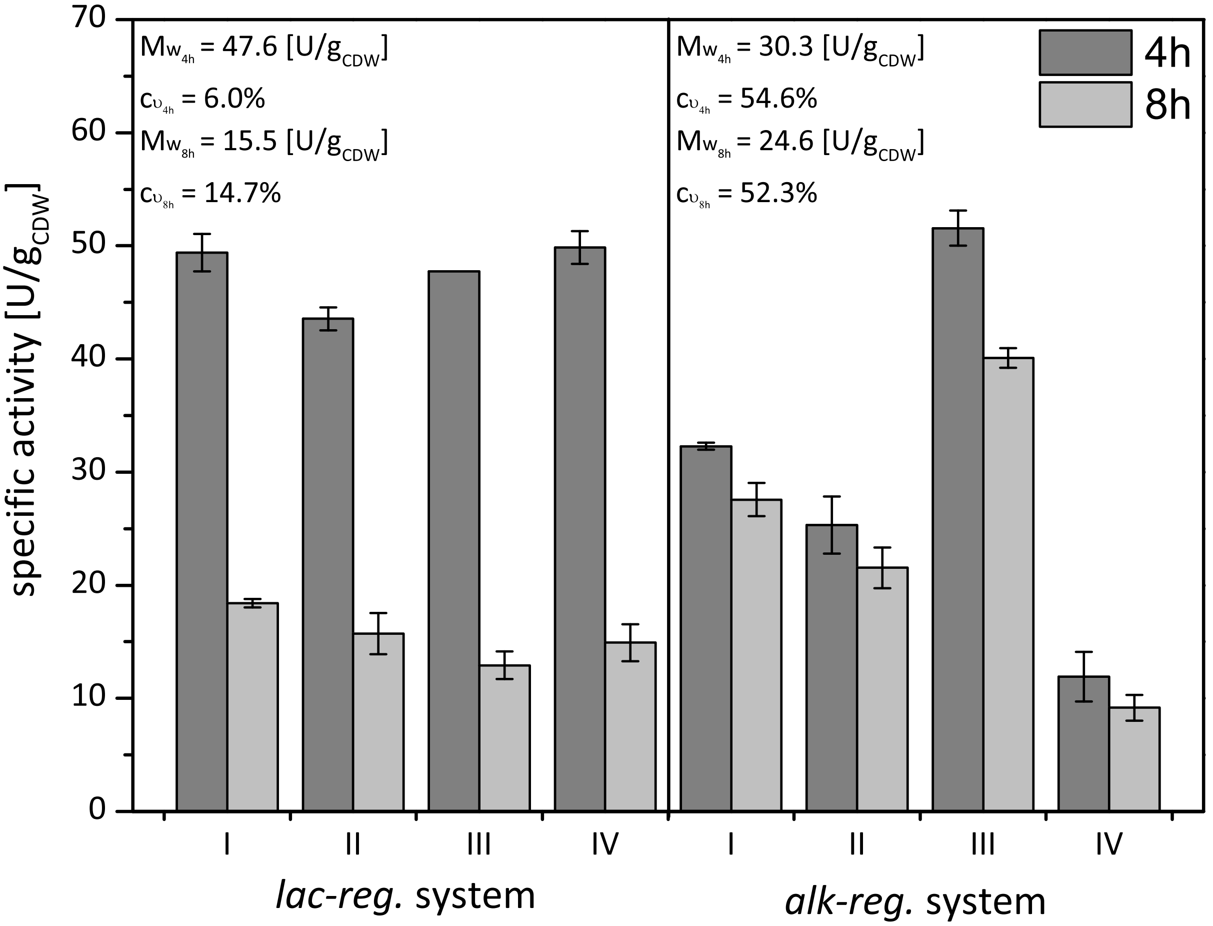

Figure S8: Specific styrene epoxidation activities of *P. putida* DOT-T1E cultures described in Figure 4 harboring plasmids encoding StyA-eGFP under the control of the *lac*- (**left**) or *alk*- (**right**) regulatory systems. After 4 of induction with either 1 mM IPTG or 0.025% (v/v) DCPK for the *lac*- and the *alk*-regulatory system, respectively, resting-cell activity assays were performed

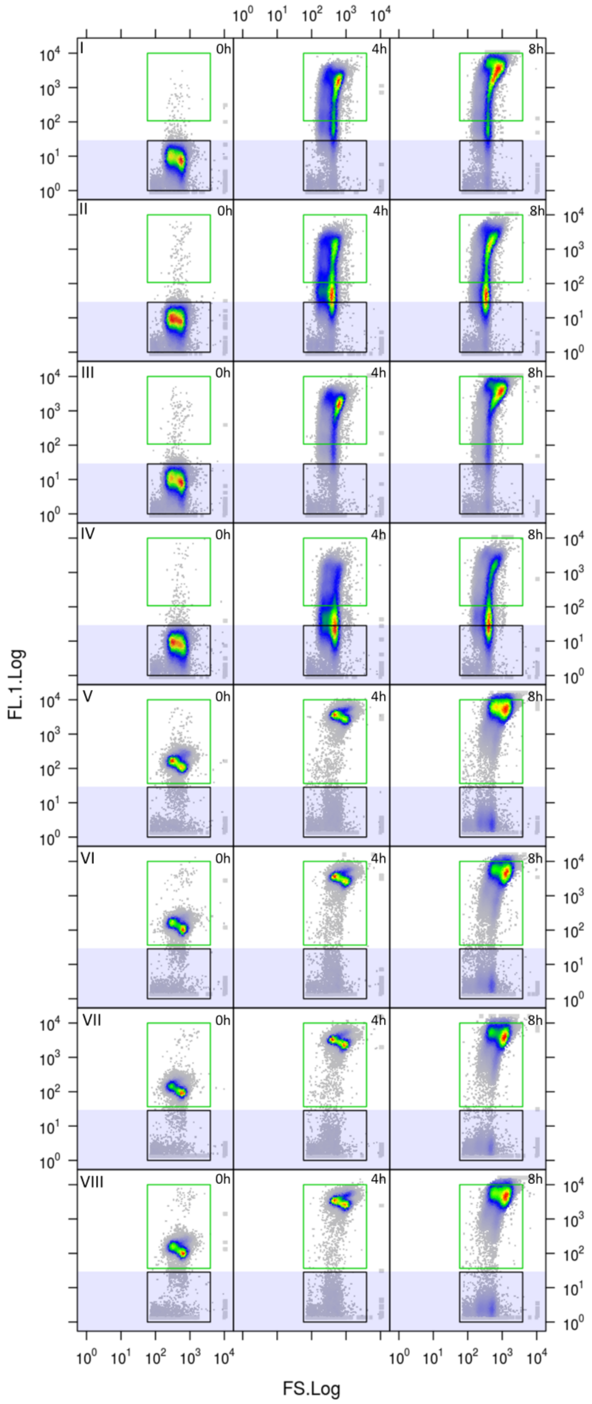

Figure S9: StyA-eGFP formation monitored via flow cytometry in the independent cultures of recombinant *P. putida* DOT-T1E (pA-EGFP_B) (**I-IV**) and *P. putida* DOT-T1E (pA-EGFP_B_lac) (**V-VIII)** described in Figure 5. X-axis, forward scatter (FS); Y-axis, eGFP fluorescence (FL). White and light-blue background marks cells judged as fluorescing (eGFP+) and non-fluorescing (eGFP−), respectively. Cells were sorted for PCN analysis using the rectangular gates depicted in green (eGFP+) and black (eGFP−).
